# Brewed Coffee and Its Components Act Through Orphan Nuclear Receptor 4A1 (NR4A1)

**DOI:** 10.3390/nu18060877

**Published:** 2026-03-10

**Authors:** Amanuel Hailemariam, Srijana Upadhyay, Arafat Rahman Oany, Wai Ning Tiffany Tsui, Vinod Srivastava, Gargi Sivaram, Kelly Churion, Robert S. Chapkin, Laurie A. Davidson, Shoshana Eitan, James J. Cai, Roger Norton, Stephen Safe

**Affiliations:** 1 Department of Veterinary Physiology and Pharmacology, College of Veterinary Medicine, Texas A&M University, College Station, TX 77843, USA; ahailemariam@tamu.edu (A.H.); arafatr@tamu.edu (A.R.O.);; 2 Department of Veterinary Integrative Biosciences, Texas A&M University, College Station, TX 77845, USA; 3 Department of Biochemistry and Biophysics, School of Veterinary Medicine and Biomedical Sciences, Texas A&M University, College Station, TX 77843, USA; gs715@tamu.edu; 4 Institute of Biosciences and Technology, College of Medicine, Texas A&M University, Houston, TX 77030, USA; 5 Department of Nutrition, Program in Integrative Nutrition and Complex Diseases, Texas A&M University, College Station, TX 77843, USA; 6 Texas A&M Regional Center of Excellence in Cancer Research, Texas A&M University, College Station, TX 77843, USA; 7 Department of Psychological and Brain Sciences, Behavioral and Cellular Neuroscience, Texas A&M University, College Station, TX 77843, USA; seitan@tamu.edu; 8 Department of Agricultural Economics, Texas A&M’s Center for Coffee Research and Education, Texas A&M University, College Station, TX 77843, USA

**Keywords:** coffee extracts, polyphenolics, NR4A1, receptor binding

## Abstract

**Background/Objective:** Coffee is the most highly consumed beverage worldwide, and coffee drinkers exhibit decreased mortality and protection from aging-related diseases. This study investigates the role of orphan nuclear receptor 4A1 (NR4A1) in mediating the effects of brewed coffee and the major polyphenolic and polyhydroxy compounds in brewed coffee and also in determining their binding to NR4A1. **Methods:** The interactions of brewed coffee and several of the major individual compounds in brewed coffee with the ligand-binding domain of NR4A1 were determined using a fluorescent binding assay. For specific compounds, binding was also carried out by surface plasmon resonance, and molecular docking studies were also performed. NR4A1-responsive Rh30 cancer cells were used as models to determine NR4A1-dependent transactivation, cell growth inhibition and inhibition of specific gene products, and in some studies, knockdown of NR4A1 by RNA interference was also determined. Inhibition of lipopolysaccharide-induced IkBα by key polyphenolics was also investigated in RAW264.7 macrophages. **Results:** Brewed coffee and several polyphenolics, including caffeic acid, ferulic acid, chlorogenic acid, *p*-coumaric acid, several cinnamic acid derivatives, kahweol, and cafestrol, bound NR4A1 in binding assays, and most Kd values were <10 µM. Brewed coffee and the major polyphenolics inhibited growth of NR4A1-responsive Rh30 cells, and this was attenuated in NR4A1-deficient Rh30 cells. These same compounds also exhibited NR4A1-dependent effects on transactivation and gene product responses in Rh30 and RAW264.7 macrophages and exhibited inverse NR4A1 agonist activity. In contrast, the NR4A1-dependent activity of caffeine and quinic acid was highly variable, suggesting that they are selective NR4A1 ligands. **Conclusions:** The results of this study demonstrate that brewed coffee and its major polyphenolics and polyhydroxy constituents are NR4A1 ligands and that NR4A1 may play an important role in the health-protective effects of coffee. These results, coupled with recent studies, indicate that NR4A1 and its ligands may play an important role in diet and health.

## 1. Introduction

There are many factors that influence the rate of aging and aging-related diseases among various populations, and in most studies, a plant-based diet enriched in fruits, nuts and vegetables is associated with decreased mortality [1,2,3]. For example, in a study of seventh-day Adventist, there was a decrease in overall mortality for vegetarians vs. non-vegetarians [4,5]. A comparison of the health status of vegetarians vs. non-vegetarians indicated that the former group had lower risk of mortality and several other health risks, including heart diseases, renal failure, infectious diseases and diabetes. However, it was estimated that Parkinson’s disease, dementia and stroke were increased in vegetarians in the older population (at 85 years of age) [4,5]. The results showing the emergence of these increased adverse health effects in older vegetarians “need further support” [4]. Coffee drinkers are a large cohort of individuals that consume an aqueous extract of roasted and ground coffee derived from the coffee bean, which is a fruit. The health impacts of coffee, the most widely consumed beverage worldwide, are similar to those observed among vegetarians and other “Blue Zone” populations with lower rates of mortality and age-related diseases [6]. Although coffee is derived from many different coffee bean varieties and there is variability in the roasting, grinding and extraction processes, there is evidence from population studies showing that coffee drinkers live longer [6]. Moreover, they are at lower risk of several age-related diseases, including metabolic disease, some cancers, Parkinson’s disease, dementia and cardiovascular diseases [7,8,9,10,11,12,13,14,15,16,17]. The remarkable health-protective effects of coffee differ from those observed in vegetarians and other long-lived “Blue Zone” populations [6], in that coffee, by weight, is only a relatively minor part of the total diet. Moreover, the relatively potent health benefits of coffee have also been observed in some therapeutic studies. For example, coffee consumption has been associated with enhanced post-diagnosis breast and colon cancer survival and a lower incidence of post-operative complications in patients recovering from colon and rectal resection surgery [18,19,20]. The effectiveness of coffee in mediating its health benefits is due to some of its more than 1000 chemical constituents, which include a large number of chemical classes and individual components, some of which are also found in vegetarian diets [21,22,23,24]. In addition to caffeine, some of the major chemicals in coffee include polyphenols and other hydroxylated compounds, including flavonoids, and these compounds exhibit antioxidant and anti-inflammatory activity which protects cells from damage and senescence [21,24,25,26,27,28,29,30].

Some of the major polyphenolics in coffee, including caffeic acid, ferulic acid and chlorogenic acid, exhibit many of the same health-protective effects associated with brewed coffee; however, their mechanisms of action are complex and not well understood [30]. Recent studies have shown that several polyphenolics, including resveratrol and flavonoids such as quercetin and kaempferol, bind orphan nuclear receptor 4A1 (NR4A1, Nur77) and exhibit activity as NR4A1 ligands capable of inhibiting endometriosis, lung cancer and rhabdomyosarcoma cell proliferation [31,32,33,34]. NR4A subfamily members were initially identified as stress-induced immediate early genes that function to attenuate stress, and results on NR4A1^−/−^ mice show that loss of NR4A1 exacerbates organ/tissue injury [35,36,37,38,39,40]. Thus, from a human health perspective, NR4A1 is a stress/inflammation-inducible gene and NR4A1/NR4A1 ligands protect against inflammation and cell/tissue damage. Thus, we hypothesize that some of the beneficial health effects of coffee may be attributed, in part, to the activity of coffee components such as NR4A1 ligands. In this study, we demonstrate that brewed coffee extracts and some of its individual polyhydroxylated constituents bind NR4A1 and exhibit NR4A1-dependent activity in cancer and non-cancer models. Since NR4A1 is pro-oncogenic in solid tumor-derived cells and regulates cell growth that is inhibited by coffee and its components, we hypothesize that these compounds are NR4A1 ligands acting as inverse agonists.

## 2. Materials and Methods

### 2.1. Cell Lines, Reagents and Transactivation Assays

Rh30 rhabdomyosarcoma cells were obtained from ATCC (Manassas, VA, USA). Cells were maintained in RPMI (St. Louis, MO, USA) medium supplemented with 10% fetal bovine serum (FBS) (Gibco/Invitrogen, Waltham, MA, USA) at 37 °C in the presence of 5% CO_2_. Cells were treated with coffee samples generously provided by Dr. Roger Norton from the Texas A&M Center for Coffee Research and Education, and these included: Honduras, Mexican, Guatemalan, El Salvadorian, and Colombian decaf coffee. Ground and espresso coffee was purchased from Polite Coffee Roasters (Bryan, TX, USA). The individual coffee compounds and related cinnamic acid derivatives (with CAS numbers) were purchased from commercial sources: *p*-coumaric acid (501-98-4), caffeine (58-08-02), ferulic acid (1135-24-6), 3,4-dimethoxycinnamic acid (2316-26-9), quinic acid (77-95-2), chlorogenic acid (327-97-9), 3-hydroxycinnamic acid (19755-02-3), 2-hydroxycinnamic acid (614-60-8), trans-cinnamic acid (140-10-3; Sigma-Aldrich, St. Louis, MO, USA), kahweol (6894-43-5), cafestrol (469-83-0; LKT Laboratories, St. Paul, MN, USA) and caffeic acid (331-39-5; Cayman Chemical Co., Ann Arbor, MI, USA). The GAL4-NR4A1 chimera ligand-binding domain (LBD) and a UAS5-luc reporter construct were transfected into cancer cells, and induction of luciferase activity was determined as previously described [31,33,34]. Antibodies used in this study included PAX3 FOX01 (PF) (C2944) and G9a (C5688515) from Cell Signaling Technologies and NR4A1 (ab283264 from Abcam (Waltham, MA, USA) and β-actin (A1978) from Sigma-Aldrich (St Louis, MO, USA).

### 2.2. Coffee Extraction

A 50 g aliquot of ground or espresso coffee was added to 250 mL of boiling water and stirred for 8–10 min. The mixture was filtered, and the aqueous extract was made up to 200 mL and stored at 2 °C until use (no freeze–thaw cycles). Each 1 mL aliquot was derived from 250 mg of ground or espresso coffee. In experiments where cells were treated with coffee extracts (1.25, 2.5, 5 and 10 µg/µL), the amount refers to weight of coffee extracted.

### 2.3. Direct Binding Assay

Fluorescence quenching experiments were conducted at 25 °C using a HORIBA Fluoromax MP 4 Fluorescence Spectrophotometer (HORIBA Instruments Incorporated, Irvine, CA, USA) to assess direct ligand binding to the NR4A1 LBD. The His-tagged LBD (His-LBD) was produced in *Escherichia coli* BL21 cells, purified, and subsequently dialyzed against phosphate-buffered saline (PBS; pH 7.4). The LBD (1.0 µM) was incubated in PBS (pH 7.4) with varying concentrations of coffee-derived ligands. Tryptophan fluorescence was recorded with an excitation wavelength of 285 nm (slit width: 5 nm), and emission spectra were collected between 300 and 420 nm (slit width: 5 nm). Data analyses were performed using Sigma Plot (version 15.1), and fluorescence intensity at 330 nm emission was quantified as a function of ligand concentration to calculate R^2^ and Kd values [32,33,34].

### 2.4. Surface Plasmon Resonance (SPR)

Surface plasmon resonance (SPR) experiments were conducted using a Biacore T200 instrument (Cytiva, Marlborough, MA, USA) operated at 25 °C. The recombinant nuclear receptor NR4A1 ligand-binding domain (LBD; molecular weight ~37 kDa) was immobilized on a CM5 sensor chip series S using amine-coupling chemistry to generate a stable, high-capacity ligand surface optimized for detection of low-molecular-weight compounds. High immobilization levels were intentionally targeted due to the inherently small mass change associated with the binding of small molecules, which produces proportionally low SPR response signals.

To promote efficient electrostatic pre-concentration of NR4A1 prior to covalent coupling, the protein was diluted to 0.75 mg/mL in 10 mM sodium acetate buffer at pH 4.5. At this pH, NR4A1 carries a net positive charge, facilitating interaction with the negatively charged carboxymethylated dextran matrix on the CM5 chip and increasing coupling efficiency. Activation of the sensor chip surface was performed using 1-ethyl-3-(3-dimethylaminopropyl) carbodiimide (EDC; 0.4 M) and *N*-hydroxysuccinimide (NHS; 0.1 M) solutions supplied by Cytiva and prepared according to the manufacturer’s Biacore T200 amine-coupling protocol. Immediately prior to injection, EDC and NHS solutions were mixed in a 1:1 (*v*/*v*) ratio, yielding effective on-chip concentrations of approximately 0.2 M EDC and 0.05 M NHS. Following ligand injection, unreacted NHS esters were quenched using 0.5 M Tris-HCl (pH 8.5) rather than ethanolamine, a modification that improved surface stability and immobilization density under these conditions. This procedure produced a final immobilization level of approximately 5000–6000 response units (RUs). A reference flow cell was prepared on the same sensor chip by subjecting it to identical EDC/NHS activation and Tris-HCl deactivation steps in the absence of protein. All experimental data were processed using double referencing, consisting of subtraction of the reference flow cell response followed by subtraction of buffer-only blank injections to remove residual systematic noise and nonspecific binding contributions. Following immobilization, the sensor surface was equilibrated in 1× PBS-P running buffer (phosphate-buffered saline containing 0.005% P20 surfactant, pH 7.4) until a stable baseline was achieved. Prior to compound analysis, the system was conditioned using five consecutive buffer injections to equilibrate the fluidics, remove residual air bubbles, and stabilize baseline response.

All analytes were prepared from 10, 20, or 25 mM DMSO stock solution and diluted into running buffer to a final 3% (*v*/*v*) DMSO concentration. Maintaining a constant DMSO concentration across all samples minimized solvent-related refractive index differences. To further correct for residual bulk refractive index effects, a DMSO solvent correction calibration curve was generated using a series of DMSO standards bracketing the 3% concentration. This solvent correction was applied to all sensorgrams prior to kinetic or equilibrium analysis, ensuring accurate alignment of response levels across injections. Compounds were serially diluted 1:2 to generate a concentration range of 0.0156–1 µM. Blank buffer injections were interspersed regularly throughout each experiment to monitor baseline stability and facilitate accurate double referencing. Each compound injection consisted of a 180 s association phase followed by a 1200 s dissociation phase at a constant flow rate of 50 µL/min, a rate selected to minimize mass-transport limitations. Due to the rapid association and dissociation kinetics observed for the majority of compounds, no regeneration step was required, allowing for multiple sequential injections without detectable loss of ligand activity or degradation of the immobilized surface.

SPR data were processed and analyzed using Biacore T200 Evaluation Software (version 3.2) (Cytiva). For compounds exhibiting rapid on/off kinetics and achieving equilibrium during the association phase, steady-state affinity analysis was performed by plotting equilibrium binding responses as a function of analyte concentration and fitting the data to a 1:1 binding isotherm to derive apparent dissociation constants (Kd). For compounds displaying resolvable association and dissociation phases, sensorgrams were globally fit using a 1:1 Langmuir binding model to obtain kinetic rate constants (Ka and Kd), from which Kd values were calculated. All reported Kd values represent mean estimates with corresponding 95% confidence intervals, calculated by nonlinear regression within Biacore Evaluation Software (version 3.2). Confidence intervals were derived from the covariance matrix of the fitted parameters and provide a quantitative measure of uncertainty associated with each affinity determination.

### 2.5. Resazurin Cell Proliferation Assay

Human Rh30 rhabdomyosarcoma cells were maintained in RPMI medium supplemented with 10% FBS and seeded into 96-well plates at a density of 2 × 10^4^ cells per well. After reaching approximately 70% confluency, cells were exposed to the indicated concentrations of brewed coffee extracts and individual compounds. Following 24 h of treatment, resazurin (0.02 mg/mL) was added to each well and incubated for 4 h. Fluorescence was then measured at 540 nm excitation and 590 nm emission to assess the reduction of resazurin to resorufin as an indicator of metabolic activity. The final DMSO concentration per well was 0.0032% to minimize solvent-related cytotoxicity. Experimental controls included both untreated cells and DMSO solvent controls.

### 2.6. Small Interfering RNA Interference Assay

Rh30 cells (2 × 10^5^) were seeded in 6-well plates and cultured for 24 h until they reached approximately 60% confluency. Transfections were performed using Lipofectamine RNAiMAX (56531) (Invitrogen, Waltham, MA, USA) according to the manufacturer’s protocol. The transfection mixture, consisting of siRNA, Lipofectamine RNAiMAX reagent, and Opti-MEM (31985-062) (Gibco, Waltham, MA, USA), was applied for 6 h, after which the medium was replaced with fresh growth medium. Cells were maintained at 37 °C with 5% CO_2_ for an additional 72 h before harvesting for protein and RNA analyses. Knockdown efficiency of NR4A1 was assessed by Western blotting using siRNAs targeting NR4A1 (siNR4A1_C) (Sigma-Aldrich, St. Louis, MO, USA). A scrambled siRNA control (CGU ACG CGG AAU ACU UCG A) (Sigma-Aldrich, St. Louis, MO, USA) was included.

### 2.7. Western Blotting

Rh30 cells (3 × 10^5^) were seeded in 6-well plates and allowed to adhere for 24 h before being treated for an additional 24 h with either DMSO (control) or varying concentrations of coffee extracts and individual compounds. Following treatment, cells were lysed with RIPA buffer (89901) (Thermo Fisher Scientific, Waltham, MA, USA) supplemented with protease (P3100) and phosphatase inhibitors (P3200) (GenDEPOT, Baker, TX, USA). Whole-cell lysates were resolved on 4–20% Mini-PROTEAN TGX gels (4561094) (Bio-Rad, Hercules, CA, USA) and transferred to polyvinylidene fluoride membranes by wet blotting. Membranes were blocked with 5% milk and subsequently incubated with primary and secondary antibodies. Protein detection was performed using Immobilon Western Chemiluminescence HRP substrates (WBKL50500) (Millipore Sigma, Burlington, MA, USA) and imaged with the Bio-Rad ChemiDoc system with the target proteins that share similar/close molecular weights; membranes were stripped with stripping buffer (46430) (Thermo Fisher Scientific, Waltham, MA, USA) and reprobed with primary and secondary antibodies. The following antibodies were utilized: PAX3-FOXO1 (C2944), G9a (C5688515), N-Myc (SC2236) (Cell Signaling Technologies, Danvers, MA, USA), and NR4A1 (ab283264) (Abcam, Waltham, MA, USA). The dilution factor for each antibody was 1:1000, except for GAPDH, for which we used 1:4000. The incubation conditions were overnight for primary antibodies and 1 h for secondary antibodies. Exposure times vary from one gel to another, ranging from 2 to 15 s. Membranes were blocked, cut accordingly with the location of target protein, and then subsequently incubated with primary and secondary antibodies.

### 2.8. Live Cell Analysis

Human Rh30 rhabdomyosarcoma cells were cultured in RPMI medium supplemented with 10% FBS and maintained under standard conditions of 37 °C in a humidified 5% CO_2_ incubator. Cells were seeded into 96-well flat-bottom plates (Corning Inc., Corning, NY, USA) at a density of 2 × 10^4^ cells per well. When cultures reached approximately 70% confluence, cells were treated with the indicated concentrations of brewed coffee extracts and selected individual compounds. Cell proliferation dynamics were monitored using the BioTek BioSpa 8 Automated Incubator and Live Cell Analysis System (Agilent Technologies, Santa Clara, CA, USA), which allows for the longitudinal kinetic imaging and automated real-time assessment of cell growth under physiologically controlled environmental conditions (temperature, humidity, and CO_2_) [40]. Cell proliferation was quantified by actual cell counts recorded every 12 h over a 24 h period. Data collection was supported by integrated imaging with the associated Cytation Cell Imaging Multi-Mode Reader (Agilent Technologies, Santa Clara, CA, USA), enabling direct visualization and quantification of cell growth parameters in situ [41,42].

### 2.9. Inhibition of NFkβ Signaling in RAW246.7 Macrophages

The RAW264.7 macrophage cell line of *Mus musculus* origin was a generous gift from Dr. Jayaraman at the Department of Chemical Engineering at Texas A&M University. Cells were maintained in 10% DMEM medium, supplemented with 10% FBS (Sigma-Aldrich) and 1% penicillin/streptomycin (Thermo Fisher Scientific, #10378016) at 5% CO_2_. Cells were seeded in 6-well plates at a density of 1 × 10^6^ cells and allowed to adhere for 24 h. Cells were treated with DMSO (negative control), cytosporone B (positive control) (Sigma Aldrich #C2997) or coffee compounds/extracts. Cells were also stimulated with lipopolysaccharide (200 ng/mL) (Sigma Aldrich, #L3012). After treatment for 1 h, lysates were extracted with RIPA Buffer (89901) (Thermo Fisher Scientific, Waltham, MA, USA) supplemented with protease (P3100) and phosphatase inhibitors (P3200) (GenDEPOT, Baker, TX, USA). Whole-cell lysates were then analyzed by Wester blotting as indicated above using a *p*-IkBα antibody (Cell Signaling, #2859P).

### 2.10. Molecular Docking Studies

The crystal structures of the human orphan nuclear receptor NR4A1 ligand-binding domain (LBD) were retrieved from the Protein Data Bank (https://www.rcsb.org; PDB IDs: 1YJE, 3V3Q, and 8Y7L) [43,44,45]. Receptor preparation was performed using Schrödinger Maestro’s Protein Preparation Wizard (Maestro, Schrödinger, LLC, New York, NY, USA), which included addition of missing hydrogen atoms, assignment of bond orders, correction of protonation states, and removal of crystallographic water molecules. Protein structures were restrained and minimized using the OPLS4 force field [46] to relieve steric clashes while preserving the overall structural integrity. All ligands selected for docking were converted into.pdbqt format using Open Babel [47]. Ligand protonation and tautomeric states were assigned using Schrödinger tools (version 2025-1) at physiological pH (7.0 ± 0.5); the most probable forms at this pH were selected for docking. Molecular docking was conducted using AutoDock Vina [48]. The docking grid was centered at coordinates X = 24.28, Y = 43.97, and Z = 23.64, with dimensions of X = 47.50 Å, Y = 36.98 Å, and Z = 54.29 Å, to fully encompass the binding site. Ligand flexibility was considered during docking, while the receptor was treated as rigid. The exhaustiveness parameter was set to 8 to ensure the adequate sampling of conformational space. Docking results were evaluated based on binding affinity, and the best poses were based on a root mean square deviation (RMSD) of 0 Å relative to the predicted binding mode. Ligand–receptor interactions were analyzed using Schrödinger Maestro Suite 14.4 (Maestro, Schrödinger, LLC, New York, NY, USA), and visualizations of docking poses were generated with PyMOL [49]. Key interactions, including hydrogen bonding, hydrophobic contacts, and π–π stacking, were examined to assess binding stability and interaction specificity as described previously [50,51,52].

### 2.11. Statistical Analysis

Statistical analyses were performed using Prism 9 software. All experiments were biological triplicates to ensure reproducibility, and results are expressed as means ± SDs. Statistical significance was determined using one-way ANOVA with Dunnett’s post hoc test, with a threshold of *p* < 0.05.

## 3. Results

**Figure 1:**
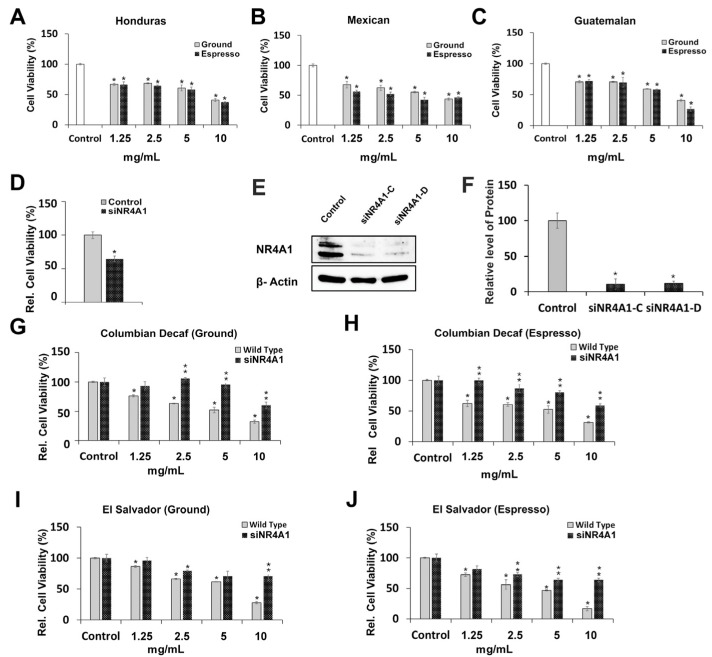
Role of NR4A1 in mediating effects of coffee extracts on Rh30 cell growth inhibition. Rh30 cells were treated with extracts of ground and espresso coffee from the Honduras (**A**), Mexico (**B**) and Guatemala (**C**). Knockdown of NR4A1 (siNR4A1) by RNA interference using 2 oligonucleotides (siNR4A1-C and siNR4A1-D) decreased Rh30 cell growth (**D**) and NR4A1 protein (**E**) in whole-cell lysates from Rh30 cells transfected with siNR4A1. Quantitation of the protein knockdown (**F**) is provided. The growth-inhibitory effects of coffee extracts were determined as outlined in the Section 2 in wild-type (NR4A1^+/+^) and NR4A1-knockdown Rh30 cells using extracts from Columbian decaf (**G**), Columbia expresso (**H**), El Salvador ground (**I**) and El Salvador (**J**) espresso coffee as outlined in the Section 2. Results are expressed as means ± SDs of 3 separate determinations (biological replicates), and significant (*p* < 0.05) growth inhibition (*) and reversal of growth inhibition by siNR4A1 (**) are indicated. In (**G**–**J**), the relative cell viability is determined with both control values set to 100%.

Ground and espresso coffee samples were obtained from commercial sources, and after extraction in boiling water for 8–10 min, the aqueous extracts were filtered. This preparation was selected to mimic the brewed process, and it is likely that other preparation methods will give extracts with different activity. The aqueous filtrates were used initially for receptor binding studies and other responses in NR4A1-responsive Rh30 Rhabdomyosarcoma cells [33,53,54]. After initial screening for cell viability, the effect of the diluted extracts [1:25 (4%); 1:50 (2%); 1:100 (1%); 1:200 (0.5%)] on the viability of Rh30 cells was determined with coffee extracts (ground and espresso) from coffee beans originating from Honduras, Mexico and Guatemala. The coffee extracts inhibited viability of Rh30 cells over all dilutions, with espresso extracts of Guatemalan coffee being more active than the ground coffee extracts but only at the highest dose (i.e., at the lowest dilution) (Figure 1A–C). Similarly, Figure 1D illustrates that knockdown of NR4A1 (siNR4A1) decreased cell viability, and Figure 1E,F confirm that siNR4A1 decreased NR4A1 protein expression. These results are comparable to previous reports showing that NR4A1 enhances Rh30 cell proliferation, which is inhibited after NR4A1 knockdown or treatment with NR4A1 ligands [53]. Columbian decaffeinated ground and espresso coffee and El Salvador ground and espresso coffee extracts also inhibited Rh30 cell viability (Figure 1G–J). The role of NR4A1 in mediating the growth-inhibitory effects of coffee extracts was further investigated in wild-type and NR4A1-deficient Rh30 cells obtained after NR4A1 knockdown by RNA interference. The effects of NR4A1 knockdown on coffee extract-mediated growth inhibition were dependent on the concentration and origin of the coffee. The most dramatic effects were observed at the highest concentration of the coffee extracts, where the differences in growth inhibition in wild-type vs. NR4A1-deficient Rh30 cells were maximal. These data obtained in NR4A1-knockdown Rh30 cells suggest that the growth-inhibitory effects of coffee extracts were due, in part, to inactivation of NR4A1, and this was comparable to the previous effects observed for bis-indole-derived NR4A1 ligands that act as inverse agonists [54].

**Figure 2:**
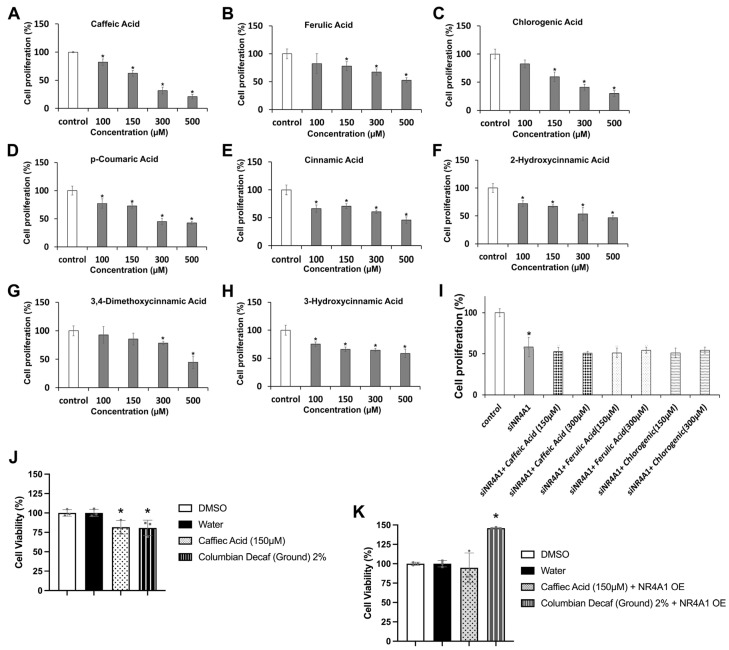
Polyphenolics inhibit Rh30 cell proliferation. Rh30 cells were treated with 100–500 µM caffeic acid (**A**), ferulic acid (**B**), chlorogenic acid (**C**), *p*-coumaric acid (**D**), cinnamic acid (**E**), 2-hydroxycinnamic acid (**F**), 3,4-dimethoxycinnamic acid (**G**), and 3-hydroxycinnamic acid (**H**) for 24 h, and effects on growth inhibition compared with DMSO (control) were determined as outlined in the Section 2. (**I**). Wild-type (NR4A1^+/+^) control and NR4A1-knockdown Rh30 cells were treated with 150 or 300 µM of the major polyphenolics in brewed coffee, and effects on cell proliferation were determined as outlined in the Section 2. Rh30 cells were treated with caffeic acid and Columbia decaf extracts, and effects on growth inhibition (**J**) and overexpression of NR4A1 (**K**) were determined as outlined in the Section 2. Results are expressed as means ± SDs of 3 separate determinations (biological replicates), and significant (*p* < 0.05) inhibition (**A**–**H**) is indicated (*). Treatment with polyphenolics did not significantly inhibit growth in NR4A1-knockdown cells.

In addition to caffeine, brewed coffee extracts express relatively high levels of chlorogenic acids and related polyphenolics, and the health benefits of coffee have been associated with these compounds and their antioxidant and anti-inflammatory activity [21,22,23,24,25,26,27,28,29]. The individual compounds included in this study are the major components of coffee (caffeine, caffeic acid, ferulic acid, chlorogenic and quinic acids), structurally related cinnamic acids, and the polyhydroxylated diterpenoids kahweol and cafestrol. Over a concentration range of 100–500 µM, the major coffee polyphenolics (caffeic acid, chlorogenic acid and ferulic acid) and also *p*-coumaric acid and related hydroxy/methoxy-substituted cinnamic acids decreased Rh30 cell viability (Figure 2A–H), and the most potent compounds at the higher concentrations were caffeic acid, chlorogenic acid and *p*-coumaric acid (Figure 2A,C,D). Moreover, in Rh30 cells in which NR4A1 had been knocked down by RNA interference, the major polyphenolics in coffee, namely, caffeic acid, chlorogenic acid and ferulic acid (150 and 300 µM) did not significantly decrease cell viability (Figure 2I). Moreover, inhibition of Rh30 cell growth by caffeic acid and decaf Columbia extract (Figure 2J) was partially reversed after overexpression of NR4A1 (Figure 2K). This indicates that similar to brewed coffee extracts, inhibition of Rh30 cell growth by major polyphenolic components in these extracts are also NR4A1-dependent; thus these components may be acting as NR4A1 inverse agonists.

**Figure 3:**
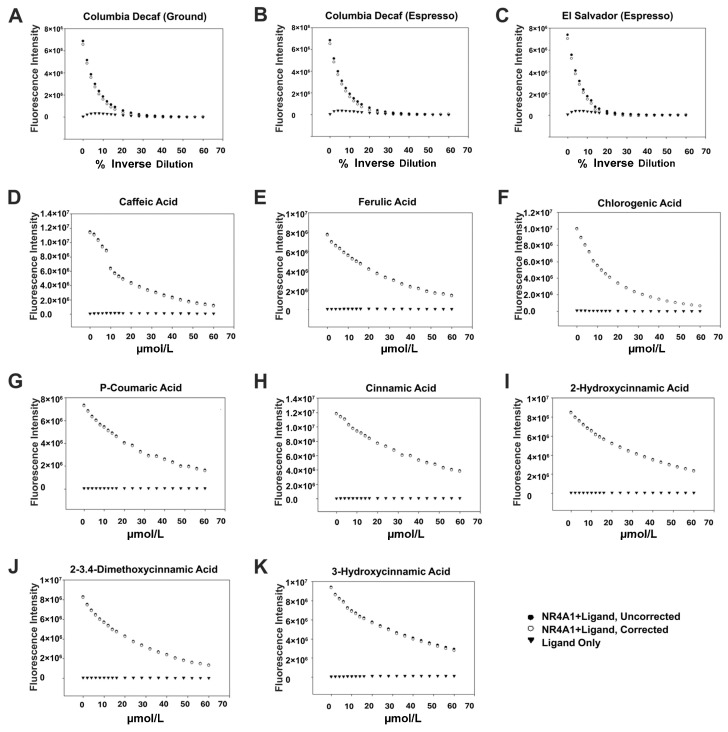
Coffee extracts and select polyphenolics bind NR4A1. Using the fluorescence quenching assay as outlined in the Section 2, the binding (K_d_ values) of brewed coffee extracts (**A**–**C**) and the following polyphenolic compounds was determined: caffeic acid (**D**), ferulic acid (**E**), chlorogenic acid (**F**), *p*-coumaric acid (**G**), cinnamic acid (**H**), 2-hydroxycinnamic acid (**I**), 3,4-dimethoxycinnamic acid (**J**) and 3-hydroxycinnamic acid (**K**). The individual polyphenolics exhibited minimal fluorescence, as well, the NR4A1 ligand and most of the corrected binding curves overlapped.

Similar to polyphenolics such as quercetin and kaempferol [31,32,33,34], the major coffee phenolics inhibit cancer cell viability, which is attenuated in NR4A1-deficient cells. Therefore, we hypothesize that coffee extracts and the major phenolics in brewed coffee bind and inactivate NR4A1. Figure 3 summarizes interactions of coffee extracts and several polyphenolics with the ligand-binding domain of NR4A1 by the fluorescent quenching of a tryptophan residue in the ligand-binding domain [33,34,43]. In these studies, we observed minimal background fluorescence by the compounds alone, and these interferences were subtracted from the total fluorescence to give the corrected value. The results show that the coffee extracts (Figure 3A–C) caffeic acid (K_d_ = 1.23 µmol/L), ferulic acid (K_d_ = 7.89 µmol/L), chlorogenic acid (K_d_ = 2.13 µmol/L), *p*-coumaric acid (K_d_ = 7.32 µmol/L), cinnamic acid (K_d_ = 6.5 µmol/L), 2-hydroxycinnamic acid (K_d_ = 9.59 µmol/L), 3,4-dimethoxycinnamic acid (K_d_ = 6.62 µmol/L) and 3-hydroxycinnamic acid (K_d_ = 6.58 µmol/L) (Figure 3D–K) bind NR4A1 and that K_d_ values for the polyphenolics range from 1.23 to 9.59 µmol/L (Table 1). Thus, in brewed coffee extracts, the polyphenolics in brewed coffee and the structurally related cinnamic acids are NR4A1 ligands that act as inverse agonists to inhibit pro-oncogenic NR4A1-mediated growth of Rh30 cells.

**Figure 4:**
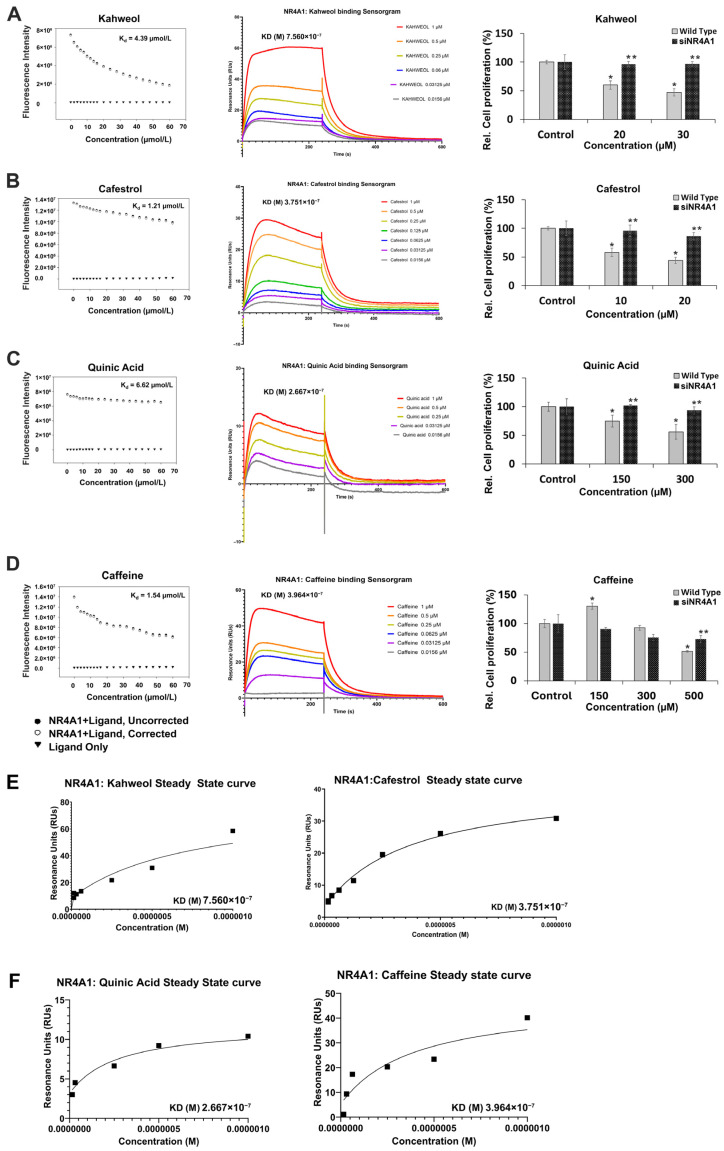
NR4A1-dependent effects of kahweol, cafestrol, quinic acid and caffeine. NR4A1 binding and NR4A1-dependent growth inhibition of kahweol (**A**), cafestrol (**B**), quinic acid (**C**) and caffeine (**D**) were determined in fluorescent quenching, SPR and cell proliferation assays as described in the Section 2. The role of NR4A1 in mediating the growth-inhibitory effects of coffee compounds was determined in Rh30 wild-type and NR4A1-knockdown cells as outlined in the Section 2. The SPR-derived equilibrium binding curves for kahweol and cafestrol (**E**), and quinic acid and caffeine (**F**) were determined as outlined in the Section 2. Results are expressed as means ± SDs of 3 separate determinations (biological replicates). Significant decreases in cell growth are indicated (*), and reversal of this effect by NR4A1 knockdown is also indicated (**). For the relative cell viability assays, both control values were set to 100%.

The two diterpenoids, kahweol and cafestrol, belong to a unique class of hydroxylated compounds that are routinely identified in brewed coffee and induce anti-inflammatory and antioxidant activity that overlaps with that induced by polyphenolics [55,56,57]. Figure 4A,B show that both kahweol and cafestrol bind NR4A1 in the fluorescence quenching assay and that their K_d_ values are 4.39 and 1.21 µM; however, because of the shallow corrected fluorescence for cafestrol, we also used SPR to determine ligand–receptor binding. The K_d_ values for SPR binding were 0.75 and 0.37 µM for kahweol and cafestrol, respectively. Both kahweol and cafestrol inhibited Rh30 cell growth, and the magnitude of the response was attenuated in Rh30 knockdown cells, suggesting a role for NR4A1 in mediating inhibition of Rh30 cell growth by kahweol and cafestrol. The K_d_ values for quinic acid (Figure 4C) and caffeine (Figure 4D) were 6.52 and 1.54 µM (fluorescence assay) and 0.26 and 0.40 µM (SPR assay), respectively; the SPR-derived equilibrium binding curves for kahweol and cafestrol (Figure 4E), and quinic acid and caffeine (Figure 4F) are also given. Both quinic acid and caffeine inhibited growth of Rh30 cells. In comparison, in NR4A1-deficient cells, growth inhibition by these compounds was slightly attenuated. These results suggest that NR4A1 may contribute to caffeine- and quinic acid-mediated inhibition of Rh30 cell growth.

**Figure 5:**
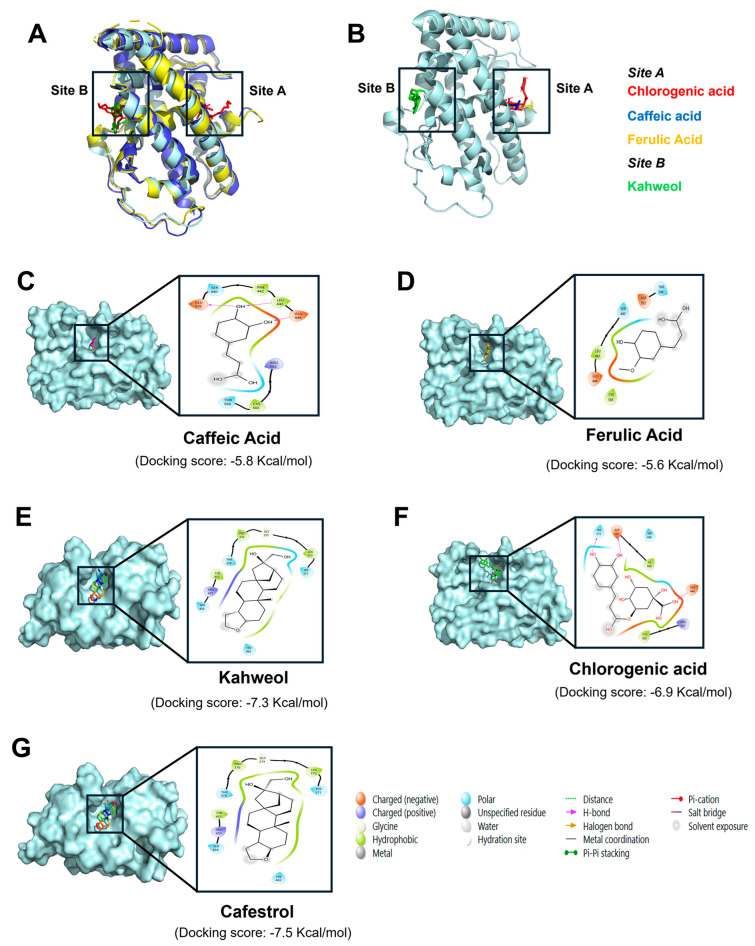
Binding of coffee compounds to the NR4A1 ligand-binding domain (LBD). (**A**). Structural superposition of NR4A1 LBD crystal structures: PDB 3V3Q (yellow), complexed with two TMPA molecules at the characteristic sites A and B; PDB 8Y7L (blue), complexed with NB1 at site B; and PDB 1YJE (cyan), used as the reference structure (target). Both 3V3Q and 8Y7L were superposed onto 1YJE with RSMD values of 0.65 and 0.87, respectively. (**B**). Kahweol was predicted to bind within site B, and caffeic acid, ferulic acid, and chlorogenic acid were localized to site A of the NR4A1 LBD crystal structure (PDB code 1YJE). (**C**–**F**). Conventional docking poses with amino acid interactions. Caffeic acid (site A) (**C**), ferulic acid (site A) (**D**), kahweol (site B) (**E**), chlorogenic acid (site A) (**F**) and cafestrol (site B) (**G**). The docking scores and interactions with sites A and B are summarized in Table 1.

To investigate the interactions of coffee-derived compounds with the NR4A1 ligand-binding domain (LBD), structural superposition of available crystal structures was performed. PDB 3V3Q (yellow), complexed with TMPA at sites A and B, and PDB 8Y7L (blue), complexed with NB1 at site B, were aligned to the reference structure PDB 1YJE (cyan). The resulting RMSD values were 0.65 Å (3V3Q) and 0.87 Å (8Y7L), confirming high structural conservation and validating 1YJE as a docking template (Figure 5A). This finding is consistent with a report showing that the NR4A1 LBD maintains a conserved fold across different ligand-bound states [45]. Docking analysis and K_d_ values for the receptor binding of the coffee components are given in Table 1, and Figure 5C–G summarize interactions of the three major polyphenolics, kahweol and cafestrol with sites A and B in the LBD of NR4A1. Docking analysis showed that caffeic acid, ferulic acid, chlorogenic acid, kahweol, and cafestrol interacted with sites A, A, A, B and B in the LBD of NR4A1, respectively. Caffeic acid formed hydrogen bonds with Glu439, Leu443, and Glu444 and polar contacts with Tyr566, Cys565, and Arg562. Ferulic acid did not form any hydrogen bonds but exhibited hydrophobic and negatively charged interactions with Glu444, Cys565, and Leu44s. In addition, interactions of chlorgenic acid with site A of NR4A1 were accompanied by hydrogen bonding (His515, Asp593, and Glu444) and hydrophobic binding (Cys565, Ile590). The functional effects of caffeine and quinic acid as NR4A1 ligands were inconsistent. However, both compounds bound NR4A1 with higher K_d_ values than the polyphenolics. Caffeine and quinic acid were predicted to bind within site A of NR4A1 but with distinct interaction profiles. Caffeine (docking score –5.1 kcal·mol^–1^) interacted mainly through electrostatic contacts with Arg562 and Arg514, along with hydrophobic interactions involving Leu443 and Leu596, without forming hydrogen bonds. In contrast, quinic acid (–5.5 kcal·mol^–1^) adopted a more polar binding mode, forming hydrogen bonds with Ser440, Arg514, Cys565, and Glu444, supported by hydrophobic contacts with Leu569 and Ile590. These results indicate that although both ligands occupy the same site, quinic acid established a more extensive interaction network than caffeine. Kahweol bound NR4A1 predominantly through hydrophobic interactions with Pro376, Thr378, His493, Tyr452, and Arg453, whereas cafestrol interacted with the same amino acid side chains as kahweol except for Tyr452. In addition, cafestrol interactions with His371, Ser454, Leu372 and Thr452 were also observed. The modeling results demonstrate that polyphenolics interact with site A whereas diterpenoids bind site B in the NR4A1 ligand-binding domain; the individual compounds that bind sites A and B exhibit differences in their interaction with various amino acid side chains.

**Figure 6:**
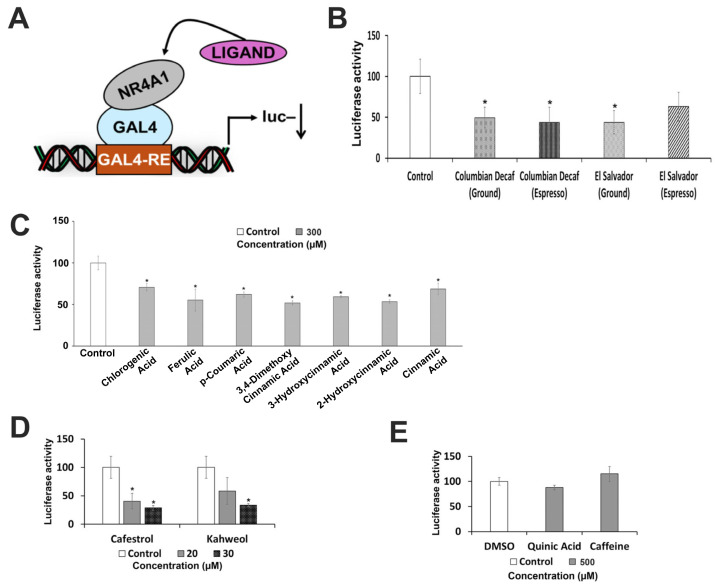
Coffee extracts and components as modulators of NR4A1-dependent transactivation. (**A**). Model of transactivation expression plasmids. Rh30 cells were transfected with GAL4-NR4A1 chimera and UAS_5_-luc expression vectors and treated with coffee extracts (**B**), polyphenolics (**C**), cafestrol and kahweol (**D**), and quinic acid and caffeine (**E**), and luciferase activity was determined as outlined in the Section 2. Results are expressed as means ± SDs of 3 separate experiments (biological replicates) for each treatment group. Significant (*p* < 0.05) inhibition is indicated (*).

Our results show that coffee extracts and some of the major polyphenolic constituents in brewed coffee inhibit Rh30 cell growth, which is attenuated in NR4A1-deficient Rh30 cells. Figure 6 shows that several coffee mixtures decrease luciferase activity in Rh30 cells transfected with a GAL4-NR4A1 (LBD) chimera and a UAS-luc reporter gene containing five GAL4 response elements (Figure 6A). The results summarized in Figure 6B,C show that the coffee mixtures and polyphenolic compounds downregulate luciferase activity, demonstrating that these compounds act as inverse agonists for this NR4A1-dependent transactivation response. Similar results are observed for cafestrol and kahweol (Figure 6D). However, neither quinic acid nor caffeine decreased or induced transactivation (Figure 6E), and this was observed over a range of doses up to 500 µM. Thus, the overall effects observed for quinic acid and caffeine with respect to their activity as NR4A1 ligands give mixed results and need to be further investigated.

**Figure 7:**
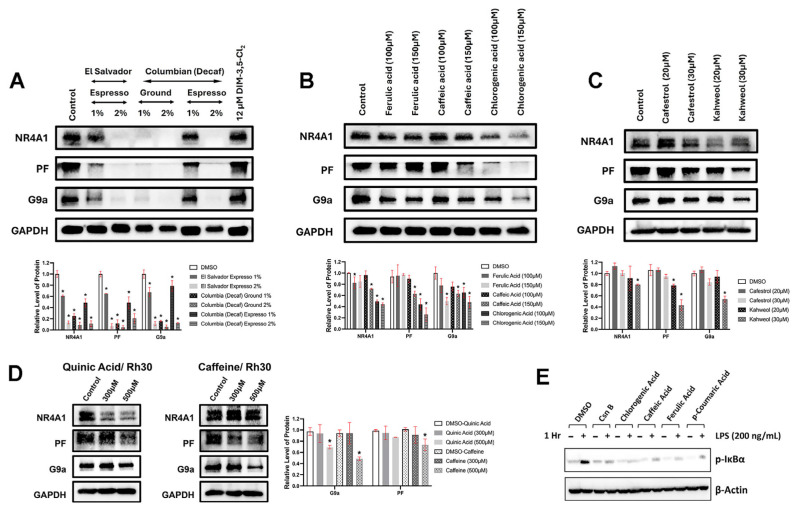
Effects of coffee extracts and individual components on NR4A1-responsive gene products. Rh30 cells were treated with brewed coffee extracts (**A**), polyphenolics (**B**), kahweol and cafestrol (**C**), and caffeine and quinic acid (**D**), and after treatment for 24 h, whole-cell lysates were analyzed by Western blots and quantitated. Results (**A**–**D**) are expressed as mean band intensities ± SDs (normalized to β-actin or GAPDH control) of 3 replicate determinations (biological replicates), and significantly (*p* < 0.05) decreased band intensities are indicated (*). (**E**). Inhibition of LPS-induced *p*-IkBα in RAW264.7 cells by polyphenolics was determined as outlined in the Section 2, and all treatments including cytosporone B (CsnB) decreased LPS-induced *p*-IkBα.

The effects of coffee extracts and individual coffee compounds were also investigated in Rh30 cells by determining their effects on two NR4A1-responsive proteins: PAX3-FOX01 (PF) and G9a [33,52,53,54]. Treatment of Rh30 cells for 24 h with selected crude coffee extracts decreased expression of G9a and PF, and the dual NR4A1/2 receptor ligand 1,1-bis(3′-indolyl)-1-(3,5-dichlorophenyl)methane (DIM-3,5-Cl_2_) also decreased expression of these NR4A1-responsive genes (Figure 7A). Individual coffee phenolics (Figure 7B) and kahweol and cafestrol (Figure 7C) also decreased expression of G9a and PF proteins. The effects of quinic acid and caffeine on the expression of PF were also determined in Rh30 cells, and both compounds decreased G9a expression, whereas only caffeine decreased PF protein (Figure 7D,E). The results for caffeine and quinic acid showed only minimal downregulation of PF and G9a (Figure 7D). The binding, transactivation, growth inhibition and Western blot results for caffeine and quinic acid suggest that both compounds are selective and relatively weak functional NR4A1 ligands. In addition, the polyphenolics ferulic acid, chlorogenic acid and caffeic acid also inhibited LPS-induced IkBα in RAW264.7 macrophages, as previously described for the NR4A1 ligand cytosporone B [58].

## 4. Discussion

The beneficial health effects of coffee consumption have been extensively investigated, and epidemiological studies show that coffee drinkers exhibit lower mortality rates and protection against multiple diseases of aging [7,8,9,10,11,12,13,14,15,16,17]. Brewed coffee from roasted beans contains multiple classes of individual compounds that contribute to the enhanced health effects of this beverage [21,22,23,24]. Polyphenolics in brewed coffee such as caffeic acid, ferulic acid and chlorogenic acids exhibit antioxidant and anti-inflammatory activity, and these compounds have been linked to the protective effects of coffee consumption [25,26,27]. Caffeine, a major component in brewed coffee, along with the polyhydroxylated cyclohexane acid derivative, quinic acid, and the dihydroxylated diterpenoids, cafestrol and kahweol, contribute to the health benefits of this beverage. Reviews on coffee extracts and their individual components report that these compounds act through diverse pathways, including direct inhibition of critical enzymes, activation of immune surveillance, modulation of the microbiome and interaction with several cell membrane and intracellular receptors [20,21,22,23,24,25,26,27,28,29,30,56,57,58]. Orphan nuclear receptor NR4A1 is an immediate early gene induced in response to inflammation and cellular stressors [26,27,28,29,30], and results of in vivo studies suggest that NR4A1 is a protective gene in maintaining cellular homeostasis in most organs and tissues and that NR4A1 ligands enhance protection and act as NR4A1 agonists (reviewed in [59]). In contrast, in transformed cells such as solid tumors, NR4A1 is overexpressed and NR4A1 ligands inhibit NR4A1-dependent pro-oncogenic activity (e.g., cell proliferation) and act as functional inverse agonists.

Many polyphenolics in coffee induce responses similar to NR4A1 ligands in non-tumor vs. tumor tissue. Like many NR4A1 ligands, the major polyphenolics in brewed coffee inhibit growth, induce apoptosis and modulate pathways and genes associated with migration and invasion of cancer cells [25,60,61,62,63]. For example, chlorogenic acid inhibited PD-L1 expression and enhanced T-cell-mediated immune surveillance in mice bearing MC38 colon cancer cells as xenografts [64]. Similar results were also obtained in breast cancer and in the same syngeneic mouse model after treatment with synthetic bis-indole-derived NR4A1 ligands [65,66]. Comparable effects were also observed using extracts containing polyphenolic compounds [66,67], suggesting that the activity of polyphenolics in cancer cells mimics some of the anticancer activities previously identified for NR4A1 ligands.

Ongoing research in several laboratories has been focused on developing ligands that interact with NR4A, and many of the small molecules that interact with NR4A1 are natural products or modified natural products. These include cytosporone B, tetrandine, resveratrol, celastrol, a bile acid metabolite and several flavonoids, some of which are found in brewed coffee [31,32,34,68,69,70,71]. The flavonoids quercetin and kaempferol are polyphenolic NR4A1 ligands that act as inverse NR4A1 agonists, inhibit cancer cell proliferation, and downregulate key pro-oncogenic genes, such as the PAX3-FOX01 and NMyc oncogenes, G9a, and several integrins, in Rh30 and other cancer cell lines [30,31,32,33,53,54,65].

Based on the overlap of the responses induced by NR4A1 ligands and polyphenolic coffee compounds, we hypothesized that brewed coffee and many individual compounds in brewed coffee exhibit activity as NR4A1 ligands. In this study, we used NR4A1-responsive Rh30 cells in a screening assay to investigate interactions of coffee/coffee polyphenolics with NR4A1. Rh30 cells were chosen for this study based on results of prior studies with NR4A1 ligands including flavonoids that demonstrate the NR4A1 responsiveness of these cells. However, the results need to be further confirmed in future studies in non-transformed tissues/organs. Aqueous extracts of various ground and expresso coffee varieties inhibited Rh30 cell growth by binding NR4A1 and downregulated luciferase activity in Rh30 cells transfected with a GAL4-NR4A1 chimera and a UAS-luc reporter gene. In NR4A1-knockdown Rh30 cells, the loss of the receptor was paralleled by decreased cytotoxicity of the coffee extracts, and this was somewhat variable depending on the source of the coffee. These results in NR4A1-responsive Rh30 cells indicate that the effects of brewed coffee are due, in part, to interactions with NR4A1.

Although some polyphenolics, including phenols such as quercetin, a minor constituent in brewed coffee, have been previously characterized as NR4A1 ligands [33,34], the binding and functional interactions of the major polyphenolic compounds in coffee with NR4A1 have not previously been determined. Our results show that the major polyphenolics in coffee, kahweol and cafestrol, bind NR4A1, inhibit NR4A1-dependent transactivation (using GAL4-NR4A/UAS-luc) and inhibit NR4A1-dependent growth and expression of NR4A1-responsive gene products in Rh30 cells. These results, coupled with molecular docking studies, suggest that the major polyphenolics in coffee (caffeic acid, ferulic acid and chlorogenic acid), kahweol, and cafestrol are NR4A1 ligands. In addition, the structurally related polyphenolics which include cinnamic acid and hydroxyl/methoxy cinnamic acids are also NR4A1 ligands, consistent with the activity of structurally related ferulic, caffeic and chlorogenic acids. Moreover, the major polyphenolics in coffee also inhibited LPS-induced *p*-IkBα in RAW264.7 cells, as previously reported for the NR4A1 ligand cytosporone B [38]. Overall, the results observed for polyphenolics, kahweol, and cafestrol suggest that these compounds are NR4A1 ligands that act as inverse NR4A1 agonists in Rh30 cells, and this parallels the effects previously observed for the bis-indole-derived compounds in this cell line [53,54]. Although both quinic acid and caffeine bind NR4A1, their functional NR4A1-dependent activity is low and variable. The role of NR4A1 in regulating the effects of caffeine and quinic acid is marginal in Rh30 cells and requires further investigation in different assays and cell lines.

A recent review showed that in mouse models of stress/inflammation-induced tissue injury, NR4A1 ligands such as cytosporone B enhanced recovery, and studies using NR4A1-knockout mice showed that the receptor was protective against tissue-induced injuries (reviewed in [59]). These results are coupled with a report showing that NR4A1-expressing C57BL/6 mice lived approximately 4 months longer than NR4A1^−/−^ mice [71], suggesting that NR4A1 may be a nutrient sensor that protects mice from aging and aging-related disease [59]. Moreover, in humans, there is evidence that NR4A1 expression decreases with age and may mimic functions of NR4A1 in mouse models [59]. Previous studies identified resveratrol and flavonoids as NR4A1 ligands, supporting a role for NR4A1 as a nutrient sensor of health-protecting chemicals in the diet [31,32,33,34]. In this study, the identification of major coffee components such as polyphenolics and the diterpenoids cafestrol and kahweol as NR4A1 ligands further supports the hypothesis that NR4A1 plays a role as a sensor of coffee-derived dietary compounds that protect from aging-related diseases [72,73]. Thus the classical anti-inflammatory effects observed for polyphenolics may be due, in part, to NR4A1-regulated responses, as previously reviewed [35,36,37,38,39,59]. Caffeine serum levels in humans are in the low µM range, and concentrations of polyphenolics are likely much lower and have not been well defined. In contrast, caffeine and polyphenolics are active in Rh30 cells at concentrations > 100 µM, indicating the overall cytotoxic effects of brewed coffee must also be due to contributions from other components in the mixture of over 1000 compounds in brewed coffee. Moreover, all of the coffee-derived polyphenolics and other coffee components act through many other pathways, and the results of this study suggest that their binding and activation of NR4A1 are only contributing factors to their overall activity and health benefits.

## 5. Conclusions

Increased coffee consumption is associated with decreased mortality rates and aging-related diseases and NR4A1 is a nutrient sensor that protects from age-related tissue/organ damage. Results of this study show that brewed coffee extracts and the major polyphenolics ferulic acid, caffeic acid, chlorogenic acid, p-coumaric acid and the diterpenoids cafestrol and kahweol bind NR4A1 and exhibit NR4A1-dependent activities. These results indicate that the health benefits derived from coffee consumption are due, in part, to their activities as NR4A1 ligands.

## Figures and Tables

**Table 1:** Individual K_d_ values and docking scores for compounds in brewed coffee.

Compound	Docking Score (kcal/mol)	K_d_ (µmol/L)		Binding Site		
		Fluor.	SPR	A	B	Other
Caffeic acid	−5.8	1.23		✓		
2,4-Dihydroxycinnamic acid	−5.4	4.96		✓		
*p*-Coumaric acid	−5.4	7.32		✓		
Quinic acid	−5.5	6.62	0.25	✓		
2-Hydroxycinnamic acid	−5.1	9.59		✓		
3-Hydroxycinnamic acid	−5.4	7.15				✓
Chlorogenic acid	−6.7	2.13		✓		
Ferulic acid	−5.6	7.89		✓		
Cinnamic acid	−5.2	6.58			✓	
Kahweol	−7.3	4.39	0.75		✓	
Cafestrol	−7.5	1.21	0.37		✓	
3,4-Dimethoxycinnamic acid	−5.5	6.62			✓	
Caffeine		1.54	0.40			

## Data Availability

Data described in the manuscript will be made available upon request pending approval by the corresponding author.

## Abbreviations

The following abbreviations are used in this manuscript:

ATCC	American Type Culture Collection
CsnB	Cytosporone B
DMSO	Dimethylsulfoxide
EDC	1-Ethyl-3-(3-dimethylaminopropyl) carbodiimide
LBD	Ligand-binding domain
LPS	Lipopolysaccharide
NHS	*N*-hydroxysuccinimide
PBS	Phosphate-buffered saline
PF	PAX3-FOX01
NR	Nuclear receptor
siRNA	Small inhibitory RNA
SPR	Surface plasmon resonance

## References

[1] Caruso C., Ligotti M.E., Accardi G., Aiello A., Duro G., Galimberti D., Candore G. (2022). How Important Are Genes to Achieve Longevity?. Int. J. Mol. Sci..

[2] Moka M.K., George M., Sriram D.K. (2024). Advancing Longevity: Exploring Antiaging Pharmaceuticals in Contemporary Clinical Trials Amid Aging Dynamics. Rejuvenation Res..

[3] Oussalah A., Levy J., Berthezène C., Alpers D.H., Guéant J.L. (2020). Health outcomes associated with vegetarian diets: An umbrella review of systematic reviews and meta-analyses. Clin. Nutr..

[4] Abris G.P., Shavlik D.J., Mathew R.O., Butler F.M., Oh J., Sirirat R., Sveen L.E., Fraser G.E. (2024). Cause-specific and all-cause mortalities in vegetarian compared with those in nonvegetarian participants from the Adventist Health Study-2 cohort. Am. J. Clin. Nutr..

[5] Santos H.D., Alabadi-Bierman A., Paalani M., Padilla S.L., Alvarez A., Beeson W.L., Fraser G.E. (2025). Living longer and lifestyle: A report on the oldest of the old in the Adventist Health Study-2. JAR Life.

[6] Buettner D. (2025). Lessons From the Blue Zones: There is No Silver Bullet (or Magic Pill) for a Long, Healthy Life. Am. J. Lifestyle Med..

[7] Oyelere A.M., Kok D.E., Bos D., Gunter M.J., Ferrari P., Keski- Rahkonen P., de Wilt J.H.W., van Halteren H.K., Kouwenhoven E.A., van Duijnhoven F.J.B. (2024). Coffee consumption is associated with a reduced risk of colorectal cancer recurrence and all-cause mortality. Int. J. Cancer.

[8] Hou C., Zeng Y., Chen W., Han X., Yang H., Ying Z., Hu Y., Sun Y., Qu Y., Fang F. (2022). Medical conditions associated with coffee consumption: Disease-trajectory and comorbidity network analyses of a prospective cohort study in UK Biobank. Am. J. Clin. Nutr..

[9] Di Maso M., Boffetta P., Negri E., La Vecchia C., Bravi F. (2021). Caffeinated Coffee Consumption and Health Outcomes in the US Population: A Dose-Response Meta-Analysis and Estimation of Disease Cases and Deaths Avoided. Adv. Nutr..

[10] Liu D., Li Z.H., Shen D., Zhang P.D., Song W.Q., Zhang W.T., Huang Q.M., Chen P.L., Zhang X.R., Mao C. (2022). Association of Sugar-Sweetened, Artificially Sweetened, and Unsweetened Coffee Consumption With All-Cause and Cause-Specific Mortality: A Large Prospective Cohort Study. Ann. Intern. Med..

[11] Doepker C., Movva N., Cohen S.S., Wikoff D.S. (2022). Benefit-risk of coffee consumption and all-cause mortality: A systematic review and disability adjusted life year analysis. Food Chem. Toxicol..

[12] Shin S., Lee J.E., Loftfield E., Shu X.O., Abe S.K., Rahman M.S., Saito E., Islam M.R., Tsugane S., Sawada N. (2022). Coffee and tea consumption and mortality from all causes, cardiovascular disease and cancer: A pooled analysis of prospective studies from the Asia Cohort Consortium. Int. J. Epidemiol..

[13] Torres-Collado L., Compañ-Gabucio L.M., González-Palacios S., Notario-Barandiaran L., Oncina-Cánovas A., Vioque J., García-de la Hera M. (2021). Coffee Consumption and All-Cause, Cardiovascular, and Cancer Mortality in an Adult Mediterranean Population. Nutrients.

[14] Park Y., Cho H., Myung S.K. (2023). Effect of Coffee Consumption on Risk of Coronary Heart Disease in a Systematic Review and Meta-Analysis of Prospective Cohort Studies. Am. J. Cardiol..

[15] Ruggiero E., Di Castelnuovo A., Costanzo S., Persichillo M., De Curtis A., Cerletti C., Donati M.B., de Gaetano G., Iacoviello L., Bonaccio M. (2021). Daily Coffee Drinking Is Associated with Lower Risks of Cardiovascular and Total Mortality in a General Italian Population: Results from the Moli-sani Study. J. Nutr..

[16] Kolb H., Martin S., Kempf K. (2021). Coffee and Lower Risk of Type 2 Diabetes: Arguments for a Causal Relationship. Nutrients.

[17] Hang D., Kværner A.S., Ma W., Hu Y., Tabung F.K., Nan H., Shen H., Mucci L.A., Chan A.T., Giovannucci E.L. (2019). Coffee consumption and plasma biomarkers of metabolic and inflammatory pathways in US health professionals. Am. J. Clin. Nutr..

[18] Farvid M.S., Spence N.D., Rosner B.A., Willett W.C., Eliassen A.H., Holmes M.D. (2021). Post-diagnostic coffee and tea consumption and breast cancer survival. Br. J. Cancer..

[19] Mackintosh C., Yuan C., Ou F.S., Zhang S., Niedzwiecki D., Chang I.W., O’Neil B.H., Mullen B.C., Lenz H.J., Blanke C.D. (2020). Association of Coffee Intake With Survival in Patients With Advanced or Metastatic Colorectal Cancer. JAMA Oncol..

[20] Piric M., Pasic F., Rifatbegovic Z., Konjic F. (2015). The Effects of Drinking Coffee While Recovering from Colon and Rectal Resection Surgery. Med. Arch..

[21] Makiso M.U., Tola Y.B., Ogah O., Endale F.L. (2023). Bioactive compounds in coffee and their role in lowering the risk of major public health consequences: A review. Food Sci. Nutr..

[22] dePaula J., Cunha S.C., Revi I., Batista A.M., Sá S.V.M.D., Calado V., Fernandes J.O., Cruz A., Farah A. (2020). Contents of key bioactive and detrimental compounds in health performance coffees compared to conventional types of coffees sold in the United States market. Food Funct..

[23] Angeloni S., Nzekoue F.K., Navarini L., Sagratini G., Torregiani E., Vittori S., Caprioli G. (2020). An analytical method for the simultaneous quantification of 30 bioactive compounds in spent coffee ground by HPLC-MS/MS. J. Mass. Spectrom..

[24] Fujioka K., Shibamoto T. (2006). Quantitation of volatiles and nonvolatile acids in an extract from coffee beverages: Correlation with antioxidant activity. J. Agric. Food Chem..

[25] Shah M.A., Faheem H.I., Hamid A., Yousaf R., Haris M., Saleem U., Shah G.M., Alhasani R.H., Althobaiti N.A., Alsharif I. (2024). The entrancing role of dietary polyphenols against the most frequent aging-associated diseases. Med. Res. Rev..

[26] Stankovic S., Mutavdzin Krneta S., Djuric D., Milosevic V., Milenkovic D. (2025). Plant Polyphenols as Heart’s Best Friends: From Health Properties, to Cellular Effects, to Molecular Mechanisms of Action. Int. J. Mol. Sci..

[27] Karagöz M.F., Koçyiğit E., Koçak T., Özturan Şirin A., Icer M.A., Ağagündüz D., Coreta-Gomes F. (2024). Decoding coffee cardiometabolic potential: Chemical composition, nutritional, and health relationships. Compr. Rev. Food Sci. Food Saf..

[28] Mirzaei S., Gholami M.H., Zabolian A., Saleki H., Farahani M.V., Hamzehlou S., Far F.B., Sharifzadeh S.O., Samarghandian S., Khan H. (2021). Caffeic acid and its derivatives as potential modulators of oncogenic molecular pathways: New hope in the fight against cancer. Pharmacol. Res..

[29] Kobylińska Z., Biesiadecki M., Kuna E., Galiniak S., Mołoń M. (2025). Coffee as a Source of Antioxidants and an Elixir of Youth. Antioxidants.

[30] Safe S., Kothari J., Hailemariam A., Upadhyay S., Davidson L.A., Chapkin R.S. (2023). Health Benefits of Coffee Consumption for Cancer and Other Diseases and Mechanisms of Action. Int. J. Mol. Sci..

[31] Zhang L., Martin G., Mohankumar K., Hampton J.T., Liu W.R., Safe S. (2022). Resveratrol Binds Nuclear Receptor 4a1 (Nr4a1) and Acts as an Nr4a1 Antagonist in Lung Cancer Cells. Mol. Pharmacol..

[32] Zhang L., Mohankumar K., Martin G., Mariyam F., Park Y., Han S.J., Safe S. (2023). Flavonoids Quercetin and Kaempferol Are NR4A1 Antagonists and Suppress Endometriosis in Female Mice. Endocrinology.

[33] Shrestha R., Mohankumar K., Martin G., Hailemariam A., Lee S.O., Jin U.H., Burghardt R., Safe S. (2021). Flavonoids kaempferol and quercetin are nuclear receptor 4A1 (NR4A1, Nur77) ligands and inhibit rhabdomyosarcoma cell and tumor growth. J. Exp. Clin. Cancer Res..

[34] Lee M., Upadhyay S., Mariyam F., Martin G., Hailemariam A., Lee K., Jayaraman A., Chapkin R.S., Lee S.O., Safe S. (2023). Flavone and Hydroxyflavones Are Ligands That Bind the Orphan Nuclear Receptor 4A1 (NR4A1). Int. J. Mol. Sci..

[35] Wang Y., Li N., Guan W., Wang D. (2025). Controversy and multiple roles of the solitary nucleus receptor Nur77 in disease and physiology. FASEB J..

[36] Hashida R., Kawabata T. (2024). Structural Perspective of NR4A Nuclear Receptor Family and Their Potential Endogenous Ligands. Biol. Pharm. Bull..

[37] Pearen M.A., Muscat G.E. (2010). Minireview: Nuclear hormone receptor 4A signaling: Implications for metabolic disease. Mol. Endocrinol..

[38] Murphy E.P., Crean D. (2015). Molecular Interactions between NR4A Orphan Nuclear Receptors and NF-κB Are Required for Appropriate Inflammatory Responses and Immune Cell Homeostasis. Biomolecules.

[39] Lith S.C., de Vries C.J.M. (2021). Nuclear receptor Nur77: Its role in chronic inflammatory diseases. Essays Biochem..

[40] Oheim M., van ‘t Hoff M., Feltz A., Zamaleeva A., Mallet J.M., Collot M. (2014). New red-fluorescent calcium indicators for optogenetics, photoactivation and multi-color imaging. Biochim Biophys Acta..

[41] Stoddart M.J. (2011). Cell viability assays: Introduction. Methods in Molecular Biology.

[42] Isherwood B., Timpson P., McGhee E.J., Anderson K.I., Canel M., Serrels A., Brunton V.G., Carragher N.O. (2011). Live cell in vitro and in vivo imaging applications: Accelerating drug discovery. Pharmaceutics..

[43] Zhan Y.Y., Chen Y., Zhang Q., Zhuang J.J., Tian M., Chen H.Z., Zhang L.R., Zhang H.K., He J.P., Wang W.J. (2012). The orphan nuclear receptor Nur77 regulates LKB1 localization and activates AMPK. Nat. Chem. Biol..

[44] Chen J., Zhao T., Hong W., Li H., Ao M., Zhong Y., Chen X., Qiu Y., Wang X., Wu Z. (2024). Discovery of a novel exceptionally potent and orally active Nur77 ligand NB1 with a distinct binding mode for cancer therapy. Acta Pharm. Sin. B.

[45] Flaig R., Greschik H., Peluso-Iltis C., Moras D. (2005). Structural basis for the cell-specific activities of the NGFI-B and the Nurr1 ligand-binding domain. J. Biol. Chem..

[46] Lu C., Wu C., Ghoreishi D., Chen W., Wang L., Damm W., Ross G.A., Dahlgren M.K., Russell E., Von Bargen C.D. (2021). OPLS4: Improving Force Field Accuracy on Challenging Regimes of Chemical Space. J. Chem. Theory Comput..

[47] O’Boyle N.M., Banck M., James C.A., Morley C., Vandermeersch T., Hutchison G.R. (2011). Open Babel: An open chemical toolbox. J. Cheminform..

[48] Trott O., Olson A.J. (2010). AutoDock Vina: Improving the speed and accuracy of docking with a new scoring function, efficient optimization, and multithreading. J. Comput. Chem..

[49] DeLano W.L. (2002). Pymol: An open-source molecular graphics tool. CCP4 Newsletter Protein Crystallogr.

[50] Oany A.R., Pervin T., Moni M.A. (2021). Pharmacoinformatics based elucidation and designing of potential inhibitors against Plasmodium falciparum to target importin α/β mediated nuclear importation. Infect. Genet. Evol..

[51] Oany A.R., Mia M., Pervin T., Junaid M., Hosen S.M.Z., Moni M.A. (2020). Design of novel viral attachment inhibitors of the spike glycoprotein (S) of severe acute respiratory syndrome coronavirus-2 (SARS-CoV-2) through virtual screening and dynamics. Int. J. Antimicrob. Agents.

[52] Upadhyay S., Hailemariam A.E., Mariyam F., Hafiz Z., Martin G., Kothari J., Farkas E., Sivaram G., Bell L., Tjalkens R. (2024). Bis-Indole Derivatives as Dual Nuclear Receptor 4A1 (NR4A1) and NR4A2 Ligands. Biomolecules.

[53] Lacey A., Hedrick E., Li X., Patel K., Doddapaneni R., Singh M., Safe S. (2016). Nuclear receptor 4A1 (NR4A1) as a drug target for treating rhabdomyosarcoma (RMS). Oncotarget.

[54] Shrestha R., Mohankumar K., Jin U.H., Martin G., Safe S. (2021). The Histone Methyltransferase Gene G9A Is Regulated by Nuclear Receptor 4A1 in Alveolar Rhabdomyosarcoma Cells. Mol. Cancer Ther..

[55] Higgins L.G., Cavin C., Itoh K., Yamamoto M., Hayes J.D. (2008). Induction of cancer chemopreventive enzymes by coffee is mediated by transcription factor Nrf2. Evidence that the coffee-specific diterpenes cafestol and kahweol confer protection against acrolein. Toxicol. Appl. Pharmacol..

[56] Rai S.P., Ansari A.H., Singh D., Singh S. (2024). Coffee, antioxidants, and brain inflammation. Progress in Brain Research.

[57] Wu K.C., McDonald P.R., Liu J., Klaassen C.D. (2014). Screening of natural compounds as activators of the keap1-nrf2 pathway. Planta Med..

[58] Patiño-Martínez E., Solís-Barbosa M.A., Santana E., González-Domínguez E., Segovia-Gamboa N.C., Meraz-Ríos M.A., Córdova E.J., Valdés J., Corbí Á.L., Sánchez-Torres C. (2022). The Nurr7 agonist Cytosporone B differentially regulates inflammatory responses in human polarized macrophages. Immunobiology.

[59] Safe S. (2025). NR4A1 Acts as a Nutrient Sensor That Inhibits the Effects of Aging. Nutrients.

[60] Lu C.H., Chen W.T., Hsieh C.H., Kuo Y.Y., Chao C.Y. (2019). Thermal cycling-hyperthermia in combination with polyphenols, epigallocatechin gallate and chlorogenic acid, exerts synergistic anticancer effect against human pancreatic cancer PANC-1 cells. PLoS ONE.

[61] Yang H., Said A.M., Huang H., Papa A.P.D., Jin G., Wu S., Ma N., Lan L., Shangguan F., Zhang Q. (2021). Chlorogenic acid depresses cellular bioenergetics to suppress pancreatic carcinoma through modulating c-Myc-TFR1 axis. Phytother. Res..

[62] Schuster C., Wolpert N., Moustaid-Moussa N., Gollahon L.S. (2022). Combinatorial Effects of the Natural Products Arctigenin, Chlorogenic Acid, and Cinnamaldehyde Commit Oxidation Assassination on Breast Cancer Cells. Antioxidants.

[63] Zeng A., Liang X., Zhu S., Liu C., Wang S., Zhang Q., Zhao J., Song L. (2021). Chlorogenic acid induces apoptosis, inhibits metastasis and improves antitumor immunity in breast cancer via the NF κB signaling pathway. Oncol. Rep..

[64] Li R., Zhan Y., Ding X., Cui J., Han Y., Zhang J., Li W., Wang L., Jiang J. (2024). Cancer Differentiation Inducer Chlorogenic Acid Suppresses PD-L1 Expression and Boosts Antitumor Immunity of PD-1 Antibody. Int. J. Biol. Sci..

[65] Karki K., Wright G.A., Mohankumar K., Jin U.H., Zhang X.H., Safe S. (2020). A Bis-Indole-Derived NR4A1 Antagonist Induces PD-L1 Degradation and Enhances Antitumor Immunity. Cancer Res..

[66] Mohankumar K., Wright G., Kumaravel S., Shrestha R., Zhang L., Abdelrahim M., Chapkin R.S., Safe S. (2023). Bis-indole-derived NR4A1 antagonists inhibit colon tumor and splenic growth and T-cell exhaustion. Cancer Immunol. Immunother..

[67] Kim A., Lee E.J., Han J.H., Chung H.S. (2024). Caryophylli Cortex Suppress PD-L1 Expression in Cancer Cells and Potentiates Anti-Tumor Immunity in a Humanized PD-1/PD-L1 Knock-In MC-38 Colon Cancer Mouse Model. Nutrients.

[68] Aru B., Güzelmeric E., Akgül A., Demirel G.Y., Kırmızıbekmez H. (2019). Antiproliferative Activity of Chemically Characterized Propolis from Turkey and Its Mechanisms of Action. Chem. Biodivers..

[69] Zhan Y., Du X., Chen H., Liu J., Zhao B., Huang D., Li G., Xu Q., Zhang M., Weimer B.C. (2008). Cytosporone B is an agonist for nuclear orphan receptor Nur77. Nat. Chem. Biol..

[70] Li W., Hang S., Fang Y., Bae S., Zhang Y., Zhang M., Wang G., McCurry M.D., Bae M., Paik D. (2021). A bacterial bile acid metabolite modulates Treg activity through the nuclear hormone receptor NR4A1. Cell Host Microbe.

[71] Hu M., Luo Q., Alitongbieke G., Chong S., Xu C., Xie L., Chen X., Zhang D., Zhou Y., Wang Z. (2017). Celastrol-Induced Nur77 Interaction with TRAF2 Alleviates Inflammation by Promoting Mitochondrial Ubiquitination and Autophagy. Mol. Cell.

[72] Yu Y., Song X., Wang X., Zheng L., Ma G., Liu W., Su H., Liu X., Liu T., Cao L. (2023). Oxidative stress impairs the Nur77-Sirt1 axis resulting in a decline in organism homeostasis during aging. Aging Cell.

[73] Chen J., Zhang Z., Liu Y., Huang L., Liu Y., Yang D., Bao X., Liu P., Ge Y., Li Q. (2024). Progressive reduction of nuclear receptor Nr4a1 mediates age-dependent cognitive decline. Alzheimer’s Dement..

